# Hydrogen Sulfide Inhibits TMPRSS2 in Human Airway Epithelial Cells: Implications for SARS-CoV-2 Infection

**DOI:** 10.3390/biomedicines9091273

**Published:** 2021-09-20

**Authors:** Giulia Pozzi, Elena Masselli, Giuliana Gobbi, Prisco Mirandola, Luis Taborda-Barata, Luca Ampollini, Paolo Carbognani, Cristina Micheloni, Francesco Corazza, Daniela Galli, Cecilia Carubbi, Marco Vitale

**Affiliations:** 1 Department of Medicine and Surgery, University of Parma, 43126 Parma, Italy; giulia.pozzi@unipr.it (G.P.); elena.masselli@unipr.it (E.M.); giuliana.gobbi@unipr.it (G.G.); prisco.mirandola@unipr.it (P.M.); luca.ampollini@unipr.it (L.A.); paolo.carbognani@unipr.it (P.C.); cristina.micheloni@unipr.it (C.M.); francesco.corazza@unipr.it (F.C.); daniela.galli@unipr.it (D.G.); marco.vitale@unipr.it (M.V.); 2 CICS-Health Sciences Research Centre, University of Beira Interior, 6201-506 Covilhã, Portugal; tabordabarata@fcsaude.ubi.pt; 3 Italian Foundation for Research in Balneotherapy (FoRST), 00198 Rome, Italy

**Keywords:** SARS-CoV-2, TMPRSS2, ACE2, hydrogen sulfide

## Abstract

The COVID-19 pandemic has now affected around 190 million people worldwide, accounting for more than 4 million confirmed deaths. Besides ongoing global vaccination, finding protective and therapeutic strategies is an urgent clinical need. SARS-CoV-2 mostly infects the host organism via the respiratory system, requiring angiotensin-converting enzyme 2 (ACE2) and transmembrane protease serine 2 (TMPRSS2) to enter target cells. Therefore, these surface proteins are considered potential druggable targets. Hydrogen sulfide (H_2_S) is a gasotransmitter produced by several cell types and is also part of natural compounds, such as sulfurous waters that are often inhaled as low-intensity therapy and prevention in different respiratory conditions. H_2_S is a potent biological mediator, with anti-oxidant, anti-inflammatory, and, as more recently shown, also anti-viral activities. Considering that respiratory epithelial cells can be directly exposed to H_2_S by inhalation, here we tested the in vitro effects of H_2_S-donors on TMPRSS2 and ACE2 expression in human upper and lower airway epithelial cells. We showed that H_2_S significantly reduces the expression of TMPRSS2 without modifying ACE2 expression both in respiratory cell lines and primary human upper and lower airway epithelial cells. Results suggest that inhalational exposure of respiratory epithelial cells to natural H_2_S sources may hinder SARS-CoV-2 entry into airway epithelial cells and, consequently, potentially prevent the virus from spreading into the lower respiratory tract and the lung.

## 1. Introduction

The pandemic viral pneumonia known as COVID-19 is caused by a novel member of the Coronaviridae family named SARS-coronavirus-2 (SARS-CoV-2).

The envelope spike (S) protein of SARS-CoV-2 plays a crucial role in coronavirus pathogenicity, promoting viral entry into target cells. The spike protein mediates receptor binding and membrane fusion on target cells [1]. Generally, the spike protein of coronaviruses is functionally divided into the S1 domain, the receptor binding domain (RBD), and the S2 domain, responsible for cell membrane fusion [2]. Virus entry requires S protein priming by cellular proteases, which induce the cleavage of S1/S2 domains and allow fusion of the viral envelope with the cellular membrane. SARS-CoV-2 engages angiotensin-converting enzyme 2 (ACE2) as the entry receptor and employs the cellular serine protease TMPRSS2 for S protein priming [3,4,5]. ACE2 and TMPRSS2 are expressed in several human cells, including cells of the respiratory tract [6,7,8]. Recent data suggest that the reduction of TMPRSS2 and/or ACE2 expression may prevent viral entry into the host cell and, consequently, infection spreading. It has been shown that ACE2-knockout is able to block SARS-CoV-2 infection of murine epithelial cells [4]. In support of this theory, Hoffmann et al. demonstrated that SARS-CoV-2 entry into bronchial epithelial cells could be prevented by the TMPRSS2 inhibitor Camostat Mesylate [3]. However, despite the global race to treat COVID-19, effective drugs and/or preventive treatments are still not available [9].

Hydrogen sulfide (H_2_S) is a colorless gas resulting from geothermal activities that can be found in vegetable proteins as well as synthetic compounds (NaHS and GYY4137) [10]. H_2_S is endogenously produced by several cells (e.g., epithelial, vascular, smooth muscle), and is mainly synthesized from L-cysteine via cytoplasmic and mitochondrial cystathionine β-synthase (CBS) and cystathionine γ-lyase (CSE) enzymes [11,12,13]. After synthesis, H_2_S acts on its molecular targets expressed by a variety of cells, including those of respiratory, cardiovascular, and neuronal systems, regulating several cellular processes [14,15,16,17]. In the respiratory tract, H_2_S participates in the regulation of important physiological functions such as airway tone, pulmonary circulation, cell proliferation, and apoptosis, locally modulating oxidative stress, inflammation, and lung fibrosis [18]. Local administration of exogenous H_2_S increases mucociliary clearance, which is beneficial by enhancing the elimination of pathogenic microorganisms [19]. Furthermore, exogenously applied H_2_S donors protect various lung damages, including acute and chronic lung injury, asthma, chronic obstructive pulmonary disease, pulmonary fibrosis, and hypoxia-induced pulmonary hypertension [20,21,22,23,24,25]. H_2_S was effective in reversing lung inflammation and improving pulmonary function in various animal models of lung injury induced by cigarette smoke, lipopolysaccharide, cerulein, hypoxia, ozone, burn, hemorrhagic shock, and infrarenal aortic cross-clamping [26,27,28,29,30,31,32,33,34,35]. Moreover, other drugs (as N-acetylcysteine and sodium thiosulfate), capable to release H_2_S, can reduce oxidative stress, inflammation, and progression of lung injury in patients with pneumonia [36,37].

A robust series of recent studies highlighted the anti-viral and anti-inflammatory activity of H_2_S in both cellular and animal models. Specifically, Respiratory Syncytial Virus (RSV) infection of airway epithelial cells has been associated with decreased CSE mRNA and protein expression, and with a reduced ability to generate intracellular H_2_S in virus-infected cells [38]. A significant increase in viral infectious particle formation has been observed in A549 cells and primary human alveolar epithelial cells treated with CSE inhibitors. Similarly, CSE knockout mice exhibited increased viral replication, lung inflammation, and enhanced clinical manifestations compared to wild type [39]. Administration of exogenous H_2_S using GYY4137, significantly reduced RSV-induced release of pro-inflammatory mediators and viral replication. GYY4137 inhibited syncytia formation and virus assembly/release and markedly improved clinical parameters and pulmonary dysfunction [39]. Consistently, H_2_S has been shown to inhibit the replication of many other RNA viruses in lungs, such as influenza A and B virus, Ebola virus, Nipah virus, human metapneumovirus, Far-eastern subtype tick-borne flavivirus, Rift Valley fever virus, and Crimean-Congo hemorrhagic fever virus [11,12,38,40]. Considering these research advances on the biology of H_2_S, a potential role against COVID-19 has been recently hypothesized [37,41,42,43,44], however, original articles with experimental data demonstrating this hypothesis have not been published so far. As a matter of fact, the effects of H_2_S on SARS-CoV-2 infection are at present completely unknown; only two reports indicate that hydrogen sulfide modulates ACE2 and TMPRSS2 expression in cellular models other than respiratory cells (not representing, therefore, the entry route for Sars-CoV-2), such as endothelial cells (in which ACE2 is up-regulated by NaHS in a murine model of atherosclerosis) and human prostate cancer cell lines (in which TMPRSS2 is down-regulated by H_2_S exposure) [45,46].

Given this background, it is reasonable to hypothesize that if H_2_S were to interfere with SARS-CoV-2 infection, it would do so by modulating the expression of SARS-CoV-2 binding receptors on target cells. In this study, we tested the effects of H_2_S donors on SARS-CoV-2 binding receptor expression in human airway epithelial cells, which is the main infection route for several respiratory viruses [47].

## 2. Materials and Methods

### 2.1. Cell Lines

BEAS-2B (human, normal SV-40 immortalized bronchial epithelial cell) and Calu-1 (human, non-small-cell lung cancer cell line) were purchased from the American Type Culture Collection (ATCC, Rockville, MD, USA). Cells were cultured in RPMI supplemented with 10% of heat-inactivated fetal bovine serum (Corning, New York City, NY, USA), 1% of penicillin and of streptomycin (Corning, New York City, NY, USA), 1% of glutamine (Euroclone, Milan, Italy, EU), and maintained at 37 °C in a water-saturated atmosphere of 5% CO_2_ in air.

For seeding and subcultivation, cells were first washed with phosphate-buffered saline (PBS) and then incubated in the presence of trypsin/EDTA solution (Euroclone, Milan, Italy, EU), until detachment.

### 2.2. Primary Epithelial Cells

For in vitro experiments on the upper airway cell model, we used human nasal epithelial primary cells (HNEpC), purchased from PromoCell (PromoCell, Heidelberg, Germany, EU). HNEpC were cultured in Airway Epithelial Cell Growth Medium (PromoCell, Heidelberg, Germany, EU) with bovine pituitary extract (0.004 mL/mL), rhEGF (10 ng/mL), insulin (recombinant human) (5 μg/mL), hydrocortisone (0.5 μg/mL), epinephrine (0.5 μg/mL), triiodo-L-thyronine (6.7 ng/mL), transferrin (recombinant human) (10 μg/mL) and retinoic acid (0.1 ng/mL), according to the manufacturer protocol, and maintained at 37 °C in a water-saturated atmosphere of 5% CO_2_ in air.

For ex-vivo experiments on lower airway cell model, we used lung tissue samples obtained from patients undergoing segmental/lobar pulmonary resections at the Thoracic Surgery Division of the University Hospital of Parma. The protocol for patient enrollment and samples collection was approved by the local Ethical Committee (study number: 682/2020/TESS/UNIPR). Lung samples were collected from 3 patients. Once removed, all specimens were cut into pieces of 0.5–0.7 cm, rinsed with cold EBSS buffer, and placed in 6-well plates in RPMI supplemented with 10% of heat-inactivated fetal bovine serum (Corning, New York City, NY, USA), 1% of penicillin and of streptomycin (Corning, New York City, NY, USA), 1% of glutamine (Euroclone, Milan, Italy, EU) and maintained at 37 °C in a water-saturated atmosphere of 5% CO_2_ in air.

### 2.3. In Vitro Cultures with H_2_S Donors

The effects of H_2_S have been tested using a fast H_2_S-releasing donor, NaHS (Sigma-Aldrich, St. Louis, MO, USA), and a slow H_2_S-releasing donor, GYY4137 (Sigma-Aldrich, St. Louis, MO, USA).

NaHS working solution was prepared just before use, diluting in RPMI a 150 mM stock solution. The working solution was immediately added to cell cultures at the final concentrations of 0.5 mM and 1 mM. Control cultures were treated as above in the absence of NaHS. GYY4137 was dissolved in DMSO at 250 mM. The working solution was prepared in complete RPMI (for BEAS-2B and Calu-1) or Airway Epithelial Cell Growth Medium (for HNEpC) and added to cell cultures at the final concentration of 0.05 mM and 0.1 mM. Control cultures were treated with DMSO diluted as above. Cells were cultured in multiwells plates in a volume of 0.5 mL/cm^2^ to ensure HS^−^ storage with low H_2_S gas escape. Cultures were kept at 37 °C, under a 5% CO_2_-enriched, H_2_O-saturated atmosphere [48,49,50,51].

Specifically, BEAS-2B and Calu-1 were treated with NaHS or GYY4137 and at 24, 48, 72 h post-treatment, cells were collected for subsequent analysis.

HNEpC were treated with NaHS 0.5 mM and 1 mM (or control) and at 24, 48 h post-treatment, cells were collected for subsequent analysis.

Immediately before collection, lung samples described above were exposed to NaHS 0.5 mM and 1 mM. At 24 and 48 hours, post-treatment samples were rinsed with EBSS and fixed with 10% formalin for 24 h for subsequent immunohistochemistry analysis. For each experimental condition, 2 pieces of lung tissue from each patient were used.

### 2.4. Analysis of Gene Expression

TMPRSS2 and ACE2 mRNA expression was tested by RNA extraction and real-time PCR assay. Specifically, RNA was extracted with the RNeasy Mini Kit (Qiagen, Frederick, MD, USA), samples with 260/280 absorbance ratio between 1.9–2 were stored at −80 °C and further used for reverse transcription.

1 μg of RNA was reverse transcribed by High-capacity RNA-to-cDNA kit (Thermo Fisher Scientific, Waltham, MA, USA) in a mix volume of 20μL and the cDNA obtained was diluted 1:3 for further real time quantitative PCR (qPCR). qPCR was performed using the Power-up SYBR Green Mastermix (Thermo Fisher Scientific, Waltham, MA, USA). The cDNAs were amplified with specific primers for TMPRSS2 (forward 5′-GTGAAAGCGGGTGTGAGGA-3′, reverse 5′-CTGTGCGGGATAGGGGTTTT-3′) and ACE2 (forward, 5′-CCACTGCTCAACTACTTTGAGCC-3′, reverse 5′-CTTATCCTCACTTTGATGCTTTGG-3′) 18S rRNA (forward, 5′-ACCGGGTTGGTTTTGATCTG-3′, reverse 5′-ATCCTGCCAGTAGCATATGC-3′) was used as a house-keeping gene for normalization of target genes, the primers were selected using the NCBI/primer-blast program (http://www.ncbi.nlm.nih.gov/tools/primer-blast/, accessed on 20 march 2020) and were synthesized by Applied Biosystem (Thermo Fisher Scientific, Waltham, MA, USA). cDNA was amplified in a final mix volume of 10 μL, 40 run cycles of amplification were performed and, as amplification conditions, we used the “default PCR thermal cycling conditions for primers with Tm ≥60 °C” reported in the Power-up SYBR Green Mastermix manufacturer protocol. All reactions were run in triplicate. Semi-quantitative analysis was based on the cycle number (Ct) at which the SYBR Green fluorescent signal crossed a threshold in the log-linear range of qPCR. The Ct values for target genes (TMPRSS2, ACE2) were normalized to the Ct value of the house-keeping gene (18S rRNA), creating ∆Ct values (Ct _target gene_ − Ct _18S rRNA_). Then the difference in ∆Ct between the treated samples and the untreated control was calculated obtaining (∆∆Ct = ∆Ct _treated_ − ∆Ct _untreated_). Finally, data were reported as the fold increase in TMPRSS2 and ACE2 mRNA in NaHS and GYY4137 treated cells compared with the untreated cells at each time point.

### 2.5. Analysis of Protein Expression

TMPRSS2 and ACE2 protein expression was tested by western blot. In brief, forty micrograms of proteins were separated on 10% SDS-polyacrylamide gel electrophoresis, transferred to nitrocellulose membrane and incubated with rabbit anti-TMPRSS2 polyclonal antibody (Abcam, Cambridge, UK, catalog number: ab92323), mouse anti-ACE2 monoclonal antibody (Thermo Fisher Scientific, Waltham, MA, USA, catalog number: (MA5-31395), respectively diluted 1:700 and 1:1000 in the blotting solution as recommended by the manufacturer protocol. Mouse anti-GAPDH monoclonal antibody (Millipore Burlington, MA, USA) was used as loading control. The anti-rabbit (Thermo Fisher Scientific, Waltham, MA, USA) or anti-mouse (Sigma-Aldrich, St. Louis, MO, USA) peroxidase-conjugated were used as secondary antibodies. Proteins were resolved by ECL SuperSignal West Pico Chemiluminescent Substrate Detection System (Thermo Fisher Scientific, Waltham, MA, USA). The densitometric analyses were performed using the ImageJ software (ImageJ bundled with 64-bit Java 1.8.0_172, NIH, USA). The O.D. values of TMPRSS2 or ACE2 were normalized to GAPDH O.D. values, for each sample. Then, the value obtained for the treated sample (NaHS 0.5–1 mM or GYY4137 0.05–0.1 mM) was normalized to the relative control condition (UNT or DMSO). The fold change in TMPRSS2 and ACE2 protein, in NaHS and GYY4137 treated cells was compared with the untreated cells at each time point.

### 2.6. Immunohistochemistry Analysis

Lung tissue samples, fixed in 10% formalin for 24 h, were embedded in paraffin [48,49,50,51,52,53]. Paraffin blocks were serially cut into 4µm sections and serial sections from each specimen were routinely stained with hematoxylin–eosin for histological examination.

Immunostaining was carried out with Vectastain Elite^®^ ABC Universal kit (Vector Laboratories, Burlingame, CA, USA) and sections were stained to detect TMPRSS2 protein by using rabbit monoclonal anti-TMPRSS2 antibody (Abcam, Cambridge, UK, catalog number: ab92323). All sections were dewaxed with xylene and rehydrated by passages through decreasing concentrations of ethanol (from 100 to 80%). To perform antigen retrieval, sections were microwaved in sodium citrate buffer (pH 6.0) at 650 W for 10 min. Endogenous peroxidase activity was then blocked by a 30 min incubation at room temperature with 3% H_2_O_2_. After rinsing in phosphate buffered saline (PBS) 0.1 M, pH 7.4 (Sigma-Aldrich, St. Louis, MO, USA), sections were pretreated for 30 min at room temperature with normal horse serum and then incubated for 60 min in a humidified dark chamber with the primary antibody (anti-TMPRSS2, 1:1000 dilution). Sections were then washed twice in PBS and incubated for 30 min with diluted biotinylated secondary antibody solution. At the end of incubation, sections were washed again in PBS and incubated for 30 min at room temperature with ABC reagent. After PBS washing, peroxidase activity was detected by incubating sections for 10–15 min with a solution of 3-3′-diaminobenzidine substrate DAB (Sigma-Aldrich, St. Louis, MO, USA) and H_2_O_2_. Sections were finally counterstained with hematoxylin solution (Sigma-Aldrich, St. Louis, MO, USA). Negative controls were treated in parallel without the primary antibody.

At least 3 labelled sections from each specimen were observed at Nikon Eclipse 80i light microscope with 20× and 40× magnification, and photomicrographs were taken using a connected Nikon Digital camera DS-U1 and NIS-Element software (Konan, Minato-ku, Tokyo).

### 2.7. Statistical Analysis

Data are presented as mean ± SD of at least 3 independent experiments. Statistical analyses were performed by using one-way analysis of variance and the Dunnet test, when applicable. Prism software (GraphPad Software Inc., San Diego, CA, USA) was used for all the calculations.

## 3. Results

### 3.1. H_2_S-Donors Reduce TMPRSS2 Expression on Airway Epithelial Cells Lines

The effects of H_2_S on TMPRSS2 and ACE2 expression were investigated in two types of airway epithelial cells: bronchial (BEAS-2B) and pulmonary (Calu-1) cell lines. Cells were cultured up to 72 h in the presence of two types (fast and slow) of H_2_S donors at different doses. Specifically, NaHS was used at concentrations of 1 mM and 0.5 mM, while GYY4137 was used at concentrations of 0.1 mM and 0.05 mM, after testing these concentrations for cell viability and cell cycle (Supplemental Figures S1–S5), that were not affected. TMPRSS2 and ACE2 expression was analyzed at 24 h, 48 h, 72 h and compared to control (UNT), for each experimental point.

**Figure 1:**
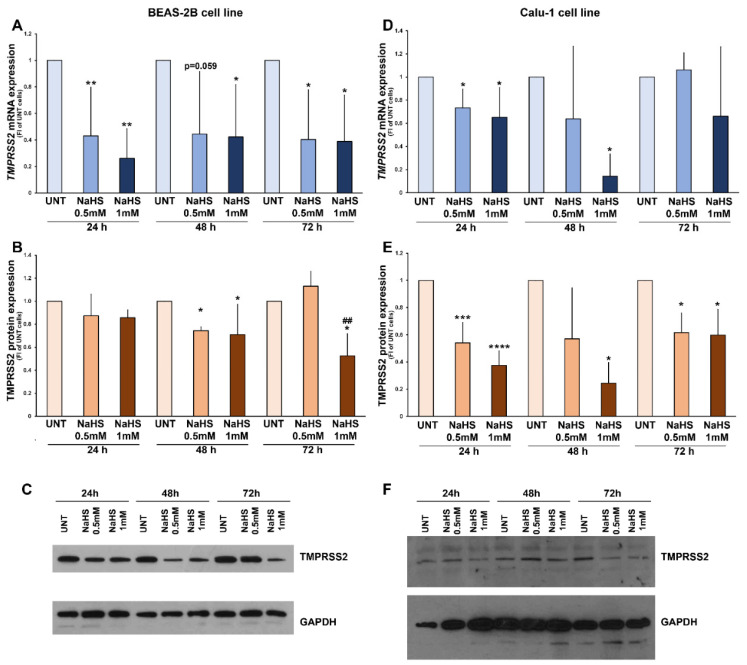
**TMPRSS2 expression in BEAS-2B and Calu-1 cells treated with NaHS.** Panels (**A**–**C**): mRNA and protein expression of TMPRSS2 in BEAS-2B cells cultured without NaHS (UNT), and in presence of NaHS (0.5–1 mM) for 24, 48, 72 h. Panels (**D**–**F**): mRNA and protein expression of TMPRSS2 in Calu-1 cells cultured without NaHS (UNT), and in presence of NaHS (0.5–1 mM) for 24, 48, 72 h. In panels (**A**,**D**), TMPRSS2 mRNA expression has been normalized to rRNA18S expression for each experimental condition, and data are reported as fold increase of UNT cells for each time point. In panels (**B**,**E**), TMPRSS2 protein expression has been normalized to GAPDH expression for each experimental condition and data are reported as fold increase of UNT cells for each time point. Data are presented as means ± SD of at least 3 independent experiments (*, *p* < 0.05; **, *p* < 0.01; ***, *p* < 0.001; ****, *p* < 0.0001; ## vs. NaHS 0.5 mM 72 h, *p* < 0.01; One-way ANOVA and Dunnett’s test). Panels (**C**,**F**): representative blots obtained with BEAS-2B and Calu-1 cells, respectively.

Both the fast and the slow-releasing H_2_S donors induced a selective down-regulation of TMPRSS2 both at the mRNA and protein levels. The fast H_2_S-releasing donor, NaHS, induced a significant down-regulation of mRNA levels of TMPRSS2 in BEAS-2B cells, which was already observed after 24 h of treatment and persisted after 48 h and 72 h (Figure 1A). Consistent with mRNA findings, at the protein level, NaHS treatment of BEAS-2B cells generated a significant down-regulation of the protease expression after 48 h, persisting at 72 h at the highest doses of NaHS (Figure 1B). The response of Calu-1 cells to NaHS treatment was similar: TMPRSS2 mRNA expression was inhibited by NaHS after 24 h and 48 h of treatment (Figure 1D), and protein expression was strongly inhibited, particularly at 24 and 48 h treatment, persisting after 72 h (Figure 1E).

**Figure 2:**
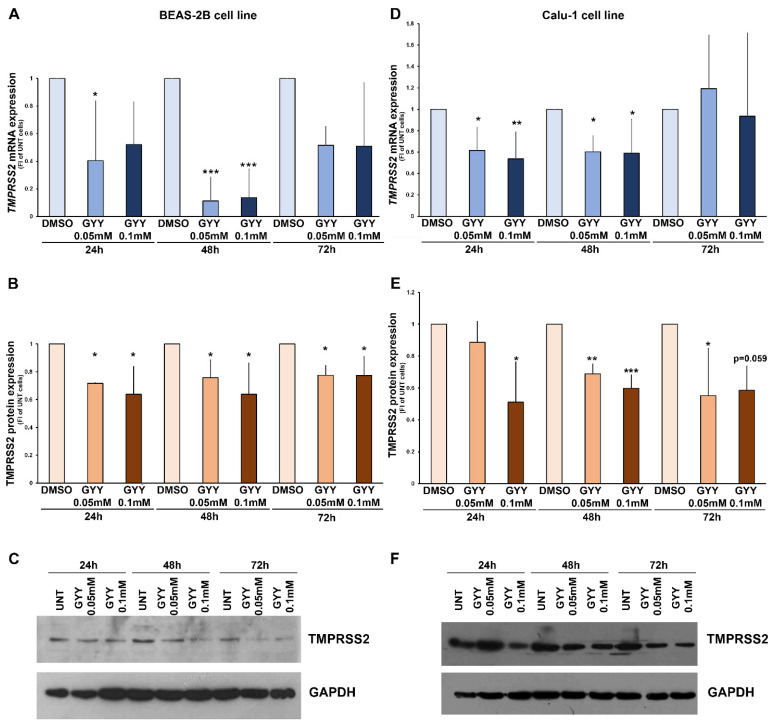
**TMPRSS2 expression in BEAS-2B and Calu-1 cells treated with GYY4137.** Panels (**A**–**C**): mRNA and protein expression of TMPRSS2 in BEAS-2B cells cultured without GYY4137 (DMSO) and in presence GYY4137 (0.05–0.1 mM) for 24, 48, 72 h. Panels (**D**–**F**): mRNA and protein expression of TMPRSS2 in Calu-1 cells cultured without GYY4137 (DMSO) and in presence GYY4137 (0.05–0.1 mM) for 24, 48, 72 h. In panels (**A**,**D**), TMPRSS2 mRNA expression has been normalized to rRNA18S expression for each experimental condition, and data are reported as fold increase of DMSO treated cells for each time point. In panels (**B**,**E**), TMPRSS2 protein expression has been normalized to GAPDH expression for each experimental condition and data are reported as fold increase of DMSO treated cells for each time point. Data are presented as mean ± SD of at least 3 independent experiments (*, *p* < 0.05; **, *p* <0.01; ***, *p* < 0.001; One-way ANOVA and Dunnett’s test). Panels (**C**,**F**): representative blots obtained with BEAS-2B and Calu-1 cells, respectively.

Similarly to NaHS, the effects of GYY4137 on TMPRSS2 were inhibitory. When BEAS-2B cells were treated with GYY4137, a strong down-regulation of TMPRSS2 mRNA expression started at 24 h, becoming stronger at 48 h of treatment, consistent with the slow release of H_2_S by GYY4137 (Figure 2A). However, GYY4137 generated a significant down-modulation of TMPRSS2 protein expression as early as 24 h treatment (Figure 2B). The response of Calu-1 cells to GYY4137 treatment was again similar to that of BEAS-2B cells (Figure 2D,E): inhibition of mRNA expression at both doses after 24 and 48 h generated an inhibition of protein expression after 24 h (at the higher dose) that became maximal for both low and high doses after 48 h treatment.

We then tested the effects of H_2_S-donors on ACE2 expression.

ACE2 mRNA expression was not modulated by NaHS or GYY4137 neither in bronchial nor in pulmonary cells (Supplementary Figure S6A–D). At the protein expression level (Supplementary Figure S6E–G), we observed an isolated reduction of ACE2 protein expression only at 48 h in Calu-1 cells treated with 1 mM of NaHS. Besides randomly scattered, this effect also appears transient, as ACE2 protein expression in NaHS treated cells was comparable to the control cells at 72 h in both Calu-1 and BEAS-2B. Moreover, GYY4137 does not affect the expression of ACE2 protein in both cell types.

Overall, these results show that the two H_2_S-donors inhibit TMPRSS2 expression on airway epithelial cell lines without significantly affecting ACE2 expression.

### 3.2. NaHS Reduces TMPRSS2 Expression on Primary Epithelial Cells of Both Upper and Lower Airways

Based on the results obtained on respiratory cell lines, we next investigated whether TMPRSS2 expression was affected by H_2_S in human primary respiratory epithelial cells from both upper and lower airways.

**Figure 3:**
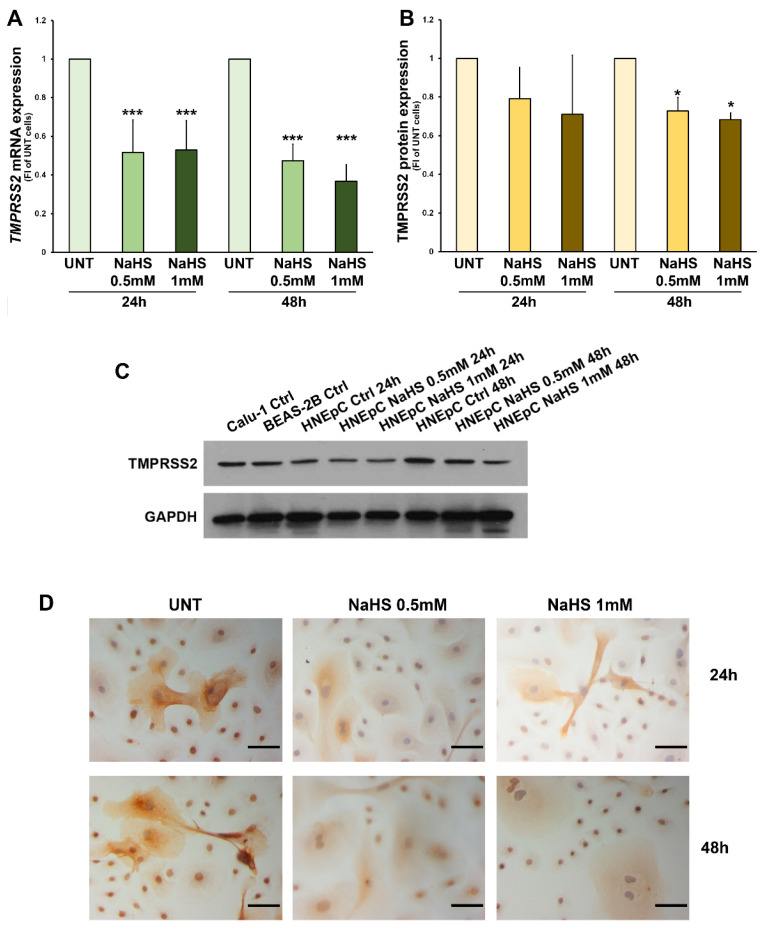
**TMPRSS2 expression in HNEpC.** Panels (**A**–**C**): mRNA and protein expression of TMPRSS2 in HNEpC cultured without NaHS (UNT), and in presence of NaHS (0.5–1 mM) for 24, 48 h. In panel (**A**), TMPRSS2 mRNA expression has been normalized to rRNA18S expression for each experimental condition, and data are reported as fold increase of UNT treated cells for each time point. In panel (**B**), TMPRSS2 protein expression has been normalized to GAPDH expression for each experimental condition and data are reported as fold increase of DMSO treated cells for each time point. Data are presented as mean ± SD of at least 3 independent experiments (*, *p* < 0.05; ***, *p* < 0.0001 One-way ANOVA and Dunnett’s test). Panel (**C**): representative blot obtained with HNEpC. Panel (**D**): immunohistochemistry analysis of TMPRSS2 in representative HNEpC cultured without NaHS (UNT), and in presence of NaHS (0.5–1 mM) for 24, 48 h; scale bar = 100 µM.

Specifically, using in vitro cultures of human nasal primary epithelial cells (HNEpC), we found that both doses of NaHS induced a strong down-regulation of TMPRSS2 mRNA level after 24 h and 48 h (Figure 3A). Western blot analysis showed a significant down-regulation of TMPRSS2 protein levels (Figure 3B) after 48 h treatment with both doses of NaHS. Immunohistochemical analysis confirmed the down-regulation of TMPRSS2 protein expression at 48 h (Figure 3C).

**Figure 4:**
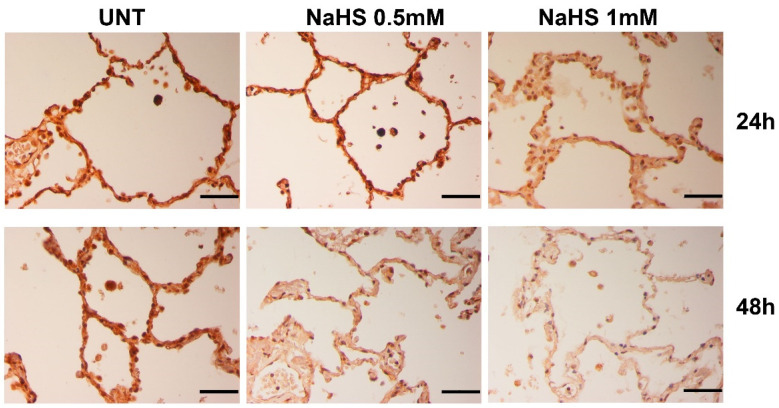
**TMPRSS2 expression in the lung.** Immunohistochemistry analysis of TMPRSS2 in a representative lung specimen incubated in RPMI medium without NaHS (UNT), and in presence of NaHS (0.5–1 mM) for 24, 48 h; scale bar = 50 µM.

The same down-regulation of TMPRSS2 expression induced by H_2_S was also detected in the primary epithelial cells of the lower respiratory tract. Indeed, ex vivo exposure of lung samples to NaHS induced a reduction of TMPRSS2 expression on human alveolar cells, especially at 48 h after treatments (Figure 4).

## 4. Discussion

Hydrogen sulfide is a gasotransmitter recognized as a biological mediator of several cellular functions and pathways. Based on its known anti-viral and anti-inflammatory effects, a number of reviews published during the last year hypothesized that H_2_S might fight COVID-19 in multiple ways [37,41,42,43,44,54,55]. Indeed, as reported by Yang: (i) H_2_S can inhibit SARS-CoV-2 replication by attenuating syncytium formation and virus assembly and release; (ii) H_2_S can protect SARS-CoV-2-induced lung damage by suppressing the immune response and inflammation development; (iii) H_2_S may block SARS-CoV-2 entry into host cells by interfering with ACE2 and TMPRSS2 [41].

Airway epithelial cells represent the first barrier between internal and external environments and the first line of defense against potentially harmful pathogens. Therefore, inhibition of virus entry and replication in the airways epithelium is of strategical importance to limit infection, allowing the onset of an efficient adaptive immune response. Hydrogen sulfide is potentially able to inhibit virus entry acting both on the virus and on the host cells. On the virus side, the RBD of SARS-CoV-2 spike protein contains disulfide bonds between cysteine residues, and it has very recently been demonstrated that the reduction of these cysteine residues by thiol-based mucolytic agents inhibits infection by coronavirus [56]. Focusing instead on the host cell side, in this work we report the first experimental evidence of the effects of H_2_S on the expression of SARS-CoV-2 binding receptors by respiratory cells. Even if the idea is not thoroughly original as it was hypothesized by other research groups in recent reviews [37,41,42,43,44], we provide the first experimental and mechanistic explanation of the protective role of H_2_S against SARS-CoV-2 infection. Indeed, we demonstrate that H_2_S-releasing donors significantly reduce the expression of the protease TMPRSS2 on epithelial cells from upper (nasal) and lower (bronco/alveolar) airways, potentially limiting viral infection. These results are consistent with the data of Zhao and co-workers that previously demonstrated NaHS significantly down-regulates TMPRSS2 mRNA levels in a prostate cancer epithelial cell line (LnCaP) [46].

H_2_S is among the most abundant compounds of natural sulfurous waters (SWs).

In general, SWs are indicated as a beneficial option for primary and secondary prevention and as part of the non-acute treatment of several respiratory conditions (e.g., chronic rhinitis, pharyngitis/laryngitis, chronic bronchitis, COPD) [10] with negligible side effects [10,57,58]. The beneficial effects of SWs reside in their ability to modulate inflammatory and immune responses, down-regulating the pro-inflammatory state [59]. In vitro studies showed that SWs can inhibit the proliferation of normal lymphocytes and T-cells obtained from patients affected by chronic immune-mediated disorders [60,61]. Also, it has been demonstrated that SWs can inhibit the release of pro-inflammatory IL-2 and INFγ from T helper-1 cells. Other studies suggested a role of H_2_S in key anti-inflammatory pathways: (i) suppression of leukocyte adherence and migration, mediated by K + ATP activation in endothelial cells and leukocytes [62]; (ii) inhibition of oxidized low-density lipoprotein-induced macrophage inflammation via NF-κB suppression [63], leading to a reduction of several pro-inflammatory cytokines (e.g., IL-1β, IL-6, and IL-8) [64]; and (iii) reduction of neutrophil toxicity by inhibition of myeloperoxidase activity [65]. Furthermore, SW can simultaneously up-regulate secretory IgA and down-regulate IgE [66]. Inhaled SWs droplets with a diameter <3μm can reach lower airways and bronchioli, while larger droplets (10μm or more) are mostly lodged in the upper airways [10]. Indeed, SW inhalation supports the recovery of lung function in cigarette smokers, decreasing nitric oxide production [67] and induces oxidative stress reduction and symptom amelioration in COPD patients [68]. For all these reasons, balneotherapy has been included in the WHO Global NCD Action Plan 2013–2023, adopted by the World Health Assembly (WHA) in 2013 [69,70], and SWs inhalations are considered a useful tool to improve respiratory health, to counteract respiratory system infections and to complement therapy of several chronic respiratory conditions [71].

Our experimental model utilizes two H_2_S-donors capable to release H_2_S at a different speed (NaHS, slow, and GYY4137, fast). In vitro systems demonstrate that the amount of free H_2_S in solution varies between 6–18% of the original donor concentration. Therefore, the amount of H_2_S in our cultures likely ranges from 5–10 μM, which is the amount that reasonably can reach the respiratory epithelia by SW inhalation. In fact, the amount of H_2_S that reaches the respiratory system during inhalational treatments depends upon various aspects which may affect particle deposition in the airways (adopted nebulizer, particle size, airway caliber, and patient’s breathing pattern) and it is generally estimated around 5–10%. Considering sulfide concentration in SW spanning from 25 µM to 2500 µM [10,72,73,74], H_2_S donor concentration used in our experiments (1000 µM and 500 µM of NaHS, 100 µM and 50 µM of GYY4137) reasonably represent the amount of H_2_S reaching the respiratory epithelia via inhalation.

Our data show that both fast- and slow-releasing H_2_S-donors induce a down-regulation of mRNA and protein expression of TMPRSS2 in bronchial and pulmonary cell lines and in primary human nasal and lung cells of healthy donors. The regulation of protease activity in the respiratory system has been previously proposed as a useful strategy to protect from virus infections [75]. Increased protease activity has been associated with increased susceptibility to respiratory viral infection. Specifically, it has been demonstrated that TMPRSS2 cellular expression enhances SARS-CoV replication; consistently, viral titers during H1N1 infection in TMPRSS2-deficient mice were significantly lower as compared to wild type animals [75]. Our data, therefore, represent the rationale for using inhalational natural H_2_S as a down-modulator of TMPRSS2 expression in respiratory cells, which spares the expression of ACE2. By contrast, Lin and colleagues reported that NaHS increases ACE2 expression in the endothelial cell line, HUVEC, and in the endothelial cell from a mouse model of atherosclerosis [45], therefore suggesting a potential H_2_S-tissue specific effect. Furthermore, ACE2 is often reported to be negatively associated with lung injury. Specifically, ACE2 activity was impaired in a mouse model of acute lung injury, and ACE2 knockout mice were more sensitive to acute lung injury as compared to normal mice [76,77]. In SARS-CoV-2 infection, ACE2 internalization would potentially result in a reduction of ACE2 activity, inducing accumulation of Angiotensin II which may exacerbate pulmonary tissue damage [78]. Hence, it is currently held that caution should be taken targeting ACE2 at a systemic level.

In summary, since: (i) inhalation of H_2_S-enriched waters has well established local anti-inflammatory and immunomodulatory effects; (ii) SWs exposure has negligible side effects and can be directly supplied to every anatomical level of the respiratory tree; (iii) H_2_S selectively down-regulates TMPRSS2 expression in airway epithelial cells; we consider all this as a proof-of-concept that H_2_S-rich inhalational treatments (i.e., by natural SWs) can contribute to the protection from SARS-CoV-2 respiratory infection, ready to be tested in a dedicated clinical trial.

## Data Availability

The data presented in this study are available on request from the corresponding author.

## Supplementary Materials

The following are available online at https://www.mdpi.com/article/10.3390/biomedicines9091273/s1, Figure S1: Cell viability on Beas-2B cell line treated with NaHS. Figure S2: Cell viability on Beas-2B cell line treated with GYY4137. Figure S3: Cell viability on Calu-1 cell line treated with NaHS. Figure S4: Cell viability on Calu-1 cell line treated with GYY4137. Figure S5: Cell cycle analysis on Beas-2B and Calu-1 cell line. Figure S6: ACE2 expression in BEAS-2B and Calu-1 cells treated with NaHS and GYY4137.

## References

[1] Li F. (2016). Structure, function, and evolution of coronavirus spike proteins. Annu. Rev. Virol..

[2] He Y., Zhou Y., Liu S., Kou Z., Li W., Farzan M., Jiang S. (2004). Receptor-binding domain of SARS-CoV spike protein induces highly potent neutralizing antibodies: Implication for developing subunit vaccine. Biochem. Biophys. Res. Commun..

[3] Hoffmann M., Kleine-Weber H., Schroeder S., Krüger N., Herrler T., Erichsen S., Schiergens T.S., Herrler G., Wu N.H., Nitsche A. (2020). SARS-CoV-2 cell entry depends on ACE2 and TMPRSS2 and is blocked by a clinically proven protease inhibitor. Cell.

[4] Zhou P., Yang X.-L., Wang X.-G., Hu B., Zhang L., Zhang W., Si H.-R., Zhu Y., Li B., Huang C.-L. (2020). A pneumonia outbreak associated with a new coronavirus of probable bat origin. Nature.

[5] Walls A.C., Park Y., Tortorici M.A., Wall A., McGuire A.T., Veesler D. (2020). Structure, function, and antigenicity of the SARS-CoV-2 spike glycoprotein. Cell.

[6] Sungnak W., Huang N., Bécavin C., Berg M., Queen R., Litvinukova M., Talavera-López C., Maatz H., Reichart D., Sampaziotis F. (2020). SARS-CoV-2 entry factors are highly expressed in nasal epithelial cells together with innate immune genes. Nat. Med..

[7] Xu H., Zhong L., Deng J., Peng J., Dan H., Zeng X., Li T., Chen Q. (2020). High expression of ACE2 receptor of 2019-nCoV on the epithelial cells of oral mucosa. Int. J. Oral Sci..

[8] Stopsack K.H., Mucci L.A., Antonarakis E.S., Nelson P.S., Kantoff P.W. (2020). TMPRSS2 and COVID-19: Serendipity or opportunity for intervention?. Cancer Discov..

[9] Ahmad I. (2021). The race to treat COVID-19: Potential therapeutic agents for the prevention and treatment of SARS-CoV-2. Eur. J. Med. Chem..

[10] Viegas J., Esteves A.F., Cardoso E.M., Arosa F.A., Vitale M., Taborda-Barata L. (2019). Biological effects of thermal water-associated hydrogen sulfide on human airways and associated immune cells: Implications for respiratory diseases. Front. Public Health.

[11] Bazhanov N., Ansar M., Ivanciuc T., Garofalo R.P., Casola A. (2017). Hydrogen sulfide: A novel player in airway development, pathophysiology of respiratory diseases, and antiviral defenses. Am. J. Respir. Cell Mol. Biol..

[12] Bazhanov N., Escaffre O., Freiberg A.N., Garofalo R.P., Casola A. (2017). Broad-range antiviral activity of hydrogen sulfide against highly pathogenic RNA viruses. Sci. Rep..

[13] Wallace J.L., Wang R. (2015). Hydrogen sulfide-based therapeutics: Exploiting a unique but ubiquitous gasotransmitter. Nat. Rev. Drug Discov..

[14] Wang R. (2002). Two’s company, three’s a crowd: Can H2S be the third endogenous gaseous transmitter?. FASEB J..

[15] Carbajo J.M., Maraver F. (2017). Sulphurous mineral waters: New applications for health. Evid.-Based Complement. Altern. Med..

[16] Benedetti F., Curreli S., Krishnan S., Davinelli S., Cocchi F., Scapagnini G., Gallo R.C., Zella D. (2017). Anti-inflammatory effects of H2S during acute bacterial infection: A review. J. Transl. Med..

[17] Benedetti F., Davinelli S., Krishnan S., Gallo R.C., Scapagnini G., Zella D., Curreli S. (2014). Sulfur compounds block MCP-1 production by Mycoplasma fermentans-infected macrophages through NF-κB inhibition. J. Transl. Med..

[18] Chen Y., Wang R. (2012). The message in the air: Hydrogen sulfide metabolism in chronic respiratory diseases. Respir. Physiol. Neurobiol..

[19] Agné A.M., Baldin J.P., Benjamin A.R., Orogo-Wenn M.C., Wichmann L., Olson K.R., Walters D.V., Althaus M. (2015). Hydrogen sulfide decreases b-adrenergic agonist-stimulated lung liquid clearance by inhibiting ENaC-mediated transepithelial sodium absorption. Am. J. Physiol.-Regul. Integr. Comp. Physiol..

[20] Zhang G., Wang P., Yang G., Cao Q., Wang R. (2013). The inhibitory role of hydrogen sulfide in airway hyperresponsiveness and inflammation in a mouse model of asthma. Am. J. Pathol..

[21] Campos D., Ravagnani F.G., Gurgueira S.A., Vercesi A.E., Teixeira S.A., Costa S.K., Muscará M.N., Ferreira H.H. (2016). Increased glutathione levels contribute to the beneficial effects of hydrogen sulfide and inducible nitric oxide inhibition in allergic lung inflammation. Int. Immunopharmacol..

[22] Madurga A., Mižíková I., Ruiz-Camp J., Vadász I., Herold S., Mayer K., Fehrenbach H., Seeger W., Morty R.E. (2014). Systemic hydrogen sulfide administration partially restores normal alveolarization in an experimental animal model of bronchopulmonary dysplasia. Am. J. Physiol.-Lung Cell. Mol. Physiol..

[23] Zhou X., An G., Chen J. (2014). Inhibitory effects of hydrogen sulphide on pulmonary fibrosis in smoking rats via attenuation of oxidative stress and inflammation. J. Cell. Mol. Med..

[24] Faller S., Seiler R., Donus R., Engelstaedter H., Hoetzel A., Spassov S.G. (2017). Pre- and post-treatment with hydrogen sulfide prevents ventilator-induced lung injury by limiting inflammation and oxidation. PLoS ONE.

[25] Feng S., Chen S., Yu W., Zhang D., Zhang C., Tang C., Du J., Jin H. (2017). H2S inhibits pulmonary arterial endothelial cell inflammation in rats with monocrotaline-induced pulmonary hypertension. Lab. Investig..

[26] Ali F.F., Abdel-Hamid H.A., Toni N.D.M. (2018). H2S attenuates acute lung inflammation induced by administration of lipopolysaccharide in adult male rats. Gen. Physiol. Biophys..

[27] Li F., Zhang P., Zhang M., Liang L., Sun X., Li M., Tang Y., Bao A., Gong J., Zhang J. (2016). Hydrogen sulfide prevents and partially reverses ozone-induced features of lung inflammation and emphysema in mice. Am. J. Respir. Cell Mol. Biol..

[28] Lin F., Liao C., Sun Y., Zhang J., Lu W., Bai Y., Liao Y., Li M., Ni X., Hou Y. (2017). Hydrogen sulfide inhibits cigarette smoke-induced endoplasmic reticulum stress and apoptosis in bronchial epithelial cells. Front. Pharmacol..

[29] Liu C.X., Tan Y.R., Xiang Y., Liu C., Liu X.A., Qin X.Q. (2018). Hydrogen sulfide protects against chemical hypoxia-induced injury via attenuation of ROS-mediated Ca^2+^ overload and mitochondrial dysfunction in human bronchial epithelial cells. Biomed. Res. Int..

[30] Meng C., Cui X., Qi S., Zhang J., Kang J., Zhou H. (2017). Lung inflation with hydrogen sulfide during the warm ischemia phase ameliorates injury in rat donor lungs via metabolic inhibition after cardiac death. Surgery.

[31] Tang B., Ma L., Yao X., Tan G., Han P., Yu T., Liu B., Sun X. (2017). Hydrogen sulfide ameliorates acute lung injury induced by infrarenal aortic cross-clamping by inhibiting inflammation and angiopoietin 2 release. J. Vasc. Surg..

[32] Vadivel A., Alphonse R.S., Ionescu L., Machado D.S., O’Reilly M., Eaton F., Haromy A., Michelakis E.D., Thébaud B. (2014). Exogenous Hydrogen sulfide (H_2_S) protects alveolar growth in experimental O_2_-induced neonatal lung injury. PLoS ONE.

[33] Wang L., Yu H., Zhang Y., Dong C., Liu B. (2017). Intravenous controlled-release hydrogen sulfide protects against ventilator-induced lung injury. Exp. Lung Res..

[34] Xu D.-Q., Gao C., Niu W., Li Y., Wang Y.-X., Gao C.-J., Ding Q., Yao L.-N., Chai W., Li Z.-C. (2013). Sodium hydrosulfide alleviates lung inflammation and cell apoptosis following resuscitated hemorrhagic shock in rats. Acta Pharmacol. Sin..

[35] Xu X., Li H., Gong Y., Zheng H., Zhao D. (2018). Hydrogen sulfide ameliorated lipopolysaccharide-induced acute lung injury by inhibiting autophagy through PI3K/Akt/mTOR pathway in mice. Biochem. Biophys. Res. Commun..

[36] Zhang Q., Ju Y., Ma Y., Wang T. (2018). *N*-acetylcysteine improves oxidative stress and inflammatory response in patients with community acquired pneumonia: A randomized controlled trial. Medicine.

[37] Evgen’Ev M.B., Frenkel A. (2020). Possible application of H_2_S-producing compounds in therapy of coronavirus (COVID-19) infection and pneumonia. Cell Stress Chaperones.

[38] Li H., Ma Y., Escaffre O., Ivanciuc T., Komaravelli N., Kelley J.P., Coletta C., Szabo C., Rockx B., Garofalo R.P. (2015). Role of hydrogen sulfide in paramyxovirus infections. J. Virol..

[39] Ivanciuc T., Sbrana E., Ansar M., Bazhanov N., Szabo C., Casola A., Garofalo R.P. (2016). Hydrogen sulfide is an antiviral and antiinflammatory endogenous gasotransmitter in the airways. Role in respiratory syncytial virus infection. Am. J. Respir. Cell Mol. Biol..

[40] Pal V.K., Bandyopadhyay P., Singh A. (2018). Hydrogen sulfide in physiology and pathogenesis of bacteria and viruses. IUBMB Life.

[41] Yang G. (2020). H_2_S as a potential defense against COVID-19?. Am. J. Physiol.-Cell Physiol..

[42] Bourgonje A.R., Offringa A.K., van Eijk L.E., Abdulle A.E., Hillebrands J.L., van der Voort P.H.J., van Goor H., van Hezik E.J. (2021). *N*-acetylcysteine and hydrogen sulfide in coronavirus disease 2019. Antioxid Redox Signal.

[43] Dattilo M. (2020). The role of host defences in Covid 19 and treatments thereof. Mol. Med..

[44] Citi V., Martelli A., Brancaleone V., Brogi S., Gojon G., Montanaro R., Morales G., Testai L., Calderone V. (2020). Anti-inflammatory and antiviral roles of hydrogen sulfide: Rationale for considering H2S donors in COVID-19 therapy. Br. J. Pharmacol..

[45] Lin Y., Zeng H., Gao L., Gu T., Wang C., Zhang H. (2017). Hydrogen sulfide attenuates atherosclerosis in a partially ligated carotid artery mouse model via regulating angiotensin converting enzyme 2 expression. Front. Physiol..

[46] Zhao K., Li S., Wu L., Lai C., Yang G. (2014). Hydrogen sulfide represses androgen receptor transactivation by targeting at the second zinc finger module. J. Biol. Chem..

[47] Zhu N., Zhang D., Wang W., Li X., Yang B., Song J., Zhao X., Huang B., Shi W., Lu R. (2020). A novel coronavirus from patients with pneumonia in China. N. Engl. J. Med..

[48] Gobbi G., Ricci F., Malinverno C., Carubbi C., Pambianco M., De Panfilis G., Vitale M., Mirandola P. (2009). Hydrogen sulfide impairs keratinocyte cell growth and adhesion inhibiting mitogen-activated protein kinase signaling. Lab. Investig..

[49] Mirandola P., Gobbi G., Sponzilli I., Pambianco M., Malinverno C., Cacchioli A., De Panfilis G., Vitale M. (2007). Exogenous hydrogen sulfide induces functional inhibition and cell death of cytotoxic lymphocytes subsets. J. Cell. Physiol..

[50] Rinaldi L., Gobbi G., Pambianco M., Micheloni C., Mirandola P., Vitale M. (2006). Hydrogen sulfide prevents apoptosis of human PMN via inhibition of p38 and caspase 3. Lab. Investig..

[51] Gobbi G., Mirandola P., Micheloni C., Solenghi E., Sponzilli I., Artico M., Soda G., Zanelli G., Pelusi G., Fiorini T. (2004). Expression of HLA class I antigen and proteasome subunits LMP-2 and LMP-10 in primary vs. metastatic breast carcinoma lesions. Int. J. Oncol..

[52] Vitale M., Pelusi G., Taroni B., Gobbi G., Micheloni C., Rezzani R., Donato F., Wang X., Ferrone S. (2005). HLA class I antigen down-regulation in primary ovary carcinoma lesions: Association with disease stage. Clin. Cancer Res..

[53] Mirandola P., Sponzilli I., Solenghi E. (2006). Down-regulation on human leukocyte antigen class I and II and beta 2-microglobulin expression in human herpesvirus-7-infected cells. J. Infect. Dis..

[54] Renieris G., Katrini K., Damoulari C., Akinosoglou K., Psarrakis C., Kyriakopoulou M., Dimopoulos G., Lada M., Koufargyris P., Giamarellos-Bourboulis E.J. (2020). Serum hydrogen sulfide and outcome association in pneumonia by the SARS-CoV-2 coronavirus. Shock.

[55] Poe F.L., Corn J. (2020). *N*-Acetylcysteine: A potential therapeutic agent for SARS-CoV-2. Med. Hypotheses.

[56] Shi Y., Zeida A., Edwards C.E., Mallory M.L., Sastre S., Machado M.R., Pickles R.J., Fu L., Liu K., Yang J. (2021). Thiol-based mucolytics exhibit antiviral activity against SARS-CoV-2 through allosteric disulfide disruption in the spike glycoprotein. bioRxiv.

[57] Keller S., König V., Mösges R. (2014). Thermal water applications in the treatment of upper respiratory tract diseases: A systematic review and meta-analysis. J. Allergy.

[58] Presta V., Ambrosini L., Carubbi C., Masselli E., Mirandola P., Arcari M.L., Gobbi G., Vitale M. (2021). Different waters for different performances: Can we imagine sport-related natural mineral spring waters?. Water.

[59] Masiero S., Maccarone M.C., Magro G. (2020). Balneotherapy and human immune function in the era of COVID-19. Int. J. Biometeorol..

[60] Costantino M., Lampa E., Nappi G. (2006). Effectiveness of sulphur spa therapy with politzer in the treatment of rhinogenic deafness. Acta Otorhinolaryngol. Ital..

[61] Salami A., Dellepiane M., Crippa B., Mora F., Guastini L., Jankowska B., Mora R. (2008). Sulphurous water inhalations in the prophylaxis of recurrent upper respiratory tract infections. Int. J. Pediatr. Otorhinolaryngol..

[62] Zanardo R.C.O., Brancaleone V., Distrutti E., Fiorucci S., Cirino G., Wallace J.L. (2006). Hydrogen sulfide is an endogenous modulator of leukocyte-mediated inflammation. FASEB J..

[63] Du J., Huang Y., Yan H., Zhang Q., Zhao M., Zhu M., Liu J., Chen S.X., Bu D., Tang C. (2014). Hydrogen sulfide suppresses oxidized low-density lipoprotein (Ox-LDL)-stimulated monocyte chemoattractant protein 1 generation from macrophages via the nuclear factor κB (NF-κB) pathway. J. Biol. Chem..

[64] Pan L.-L., Liu X.-H., Gong Q., Wu D., Zhu Y.-Z. (2011). Hydrogen sulfide attenuated tumor necrosis factor-α-induced inflammatory signaling and dysfunction in vascular endothelial cells. PLoS ONE.

[65] Pálinkás Z., Furtmüller P.G., Nagy A., Jakopitsch C., Pirker K.F., Magierowski M., Jasnos K., Wallace J.L., Obinger C., Nagy P. (2014). Interactions of hydrogen sulfide with myeloperoxidase. Br. J. Pharmacol..

[66] Pane G., Gaggero G., Mora F. (2011). Recent results about antioxidant and immunomodulatory effects of sulphurous water. J. Biol. Res..

[67] Carubbi C., Masselli E., Calabrò E., Bonati E., Galeone C., Andreoli R., Goldoni M., Corradi M., Sverzellati N., Pozzi G. (2019). Sulphurous thermal water inhalation impacts respiratory metabolic parameters in heavy smokers. Int. J. Biometeorol..

[68] Contoli M., Gnesini G., Forini G., Marku B., Pauletti A., Padovani A., Casolari P., Taurino L., Ferraro A., Chicca M. (2013). Reducing agents decrease the oxidative burst and improve clinical outcomes in COPD patients: A randomised controlled trial on the effects of sulphurous thermal water inhalation. Sci. World J..

[69] World Health Organization (2013). Global Action Plan for the Prevention and Control of Noncommunicable Diseases 2013–2020. http://www.who.int/nmh/events/ncd_action_plan/en/.

[70] World Health Organization (2013). World Health Assembly Resolution WHA66.10. Follow-Up to the Political Declaration of the High-Level Meeting of the General Assembly on the Prevention and Control of Non-Communicable Diseases. http://apps.who.int/gb/ebwha/pdf_files/WHA66/A66_R10-en.pdf?ua=1.

[71] Khaltaev N., Solimene U., Vitale F., Zanasi A. (2020). Balneotherapy and hydrotherapy in chronic respiratory disease. J. Thorac. Dis..

[72] Marotta D., Sica C. (1993). Composizione e classificazione delle acque minerali italiane. Ann. Chim. Appl..

[73] Braga P.C., Sambataro G., Sasso M.D., Culici M., Alfieri M., Nappi G. (2007). Antioxidant effect of sulphurous thermal water on human neutrophil bursts: Chemiluminescence evaluation. Respiration.

[74] Gambari L., Grigolo B., Filardo G., Grassi F. (2020). Sulfurous thermal waters stimulate the osteogenic differentiation of human mesenchymal stromal cells—An in vitro study. Biomed. Pharmacother..

[75] Meyer M., Jaspers I. (2015). Respiratory protease/antiprotease balance determines susceptibility to viral infection and can be modified by nutritional antioxidants. Am. J. Physiol.-Lung Cell. Mol. Physiol..

[76] Imai Y., Kuba K., Rao S., Huan Y., Guo F., Guan B., Yang P., Sarao R., Wada T., Leong-Poi H. (2005). Angiotensin-converting enzyme 2 protects from severe acute lung failure. Nature.

[77] Kuba K., Imai Y., Penninger J.M. (2006). Angiotensin-converting enzyme 2 in lung diseases. Curr. Opin. Pharmacol..

[78] Abassi Z., Assady S., Khoury E.E., Heyman S.N. (2020). Letter to the editor: Angiotensin-converting enzyme 2: An ally or a trojan horse? Implications to SARS-CoV-2-related cardiovascular complications. Am. J. Physiol.-Heart Circ. Physiol..

