# Supplementary material for: Hydrogen Sulfide Inhibits TMPRSS2 in Human Airway Epithelial Cells: Implications for SARS-CoV-2 Infection
Source: Biomedicines. 2021 Sep 20;9(9):1273. doi: 10.3390/biomedicines9091273 (PMC8469712; doi:10.3390/biomedicines9091273)
Supplement: Supplementary file 1 [file biomedicines-09-01273-s001.zip › biomedicines-1336841-supplementary.pdf]

Supplemetal material of:

Hydrogen Sulfide Inhibits TMPRSS2 in human airway epithelial cells: implications for SARS-CoV-2 infection

Giulia Pozzi, Elena Masselli, Giuliana Gobbi, Prisco Mirandola, Luis Taborda-Barata, Luca Ampollini, Paolo Carbognani, Cristina Micheloni, Francesco Corazza, Daniela Galli, Cecilia Carubbi and Marco Vitale[1].

1. *Material and Methods*

Flow cytometry

The effects of H2S on cell viability and cell cycle of BEAS-2B and Calu-1 cells, were investigated by flow cytometry. Specifically, for cell viability, culture medium of cells (likely containing dead cells) was collected, adherent cells were trypsinized, added to the previously collected culture medium and centrifugated at 1200 rpm for 8′. Supernatant was discarded and the cell pellet was washed with PBS and centrifugated at 1200 rpm for 8′. Supernatant was discarded and the cell pellet was incubated with PBS containing 2.5 μg/mL propidium iodide for 15′ in dark room temperature before analysis. Analysis of samples was performed by CytoFLEX flow cytometer (Beckman Coulter).

For cell cycle analysis BEAS-2B and Calu-1 cells were trypsinized, washed with PBS and permeabilized with 70% ethanol for 1h at 4°C. The cells were washed with PBS and incubated with PBS containing 20 μg/mL propidium iodide (PI) and 100 μg/mL RNAse-A for 15′ in dark room temperature before analysis.

Analysis of samples was performed by CytoFLEX flow cytometer (Beckman Coulter).

2. *Figures*

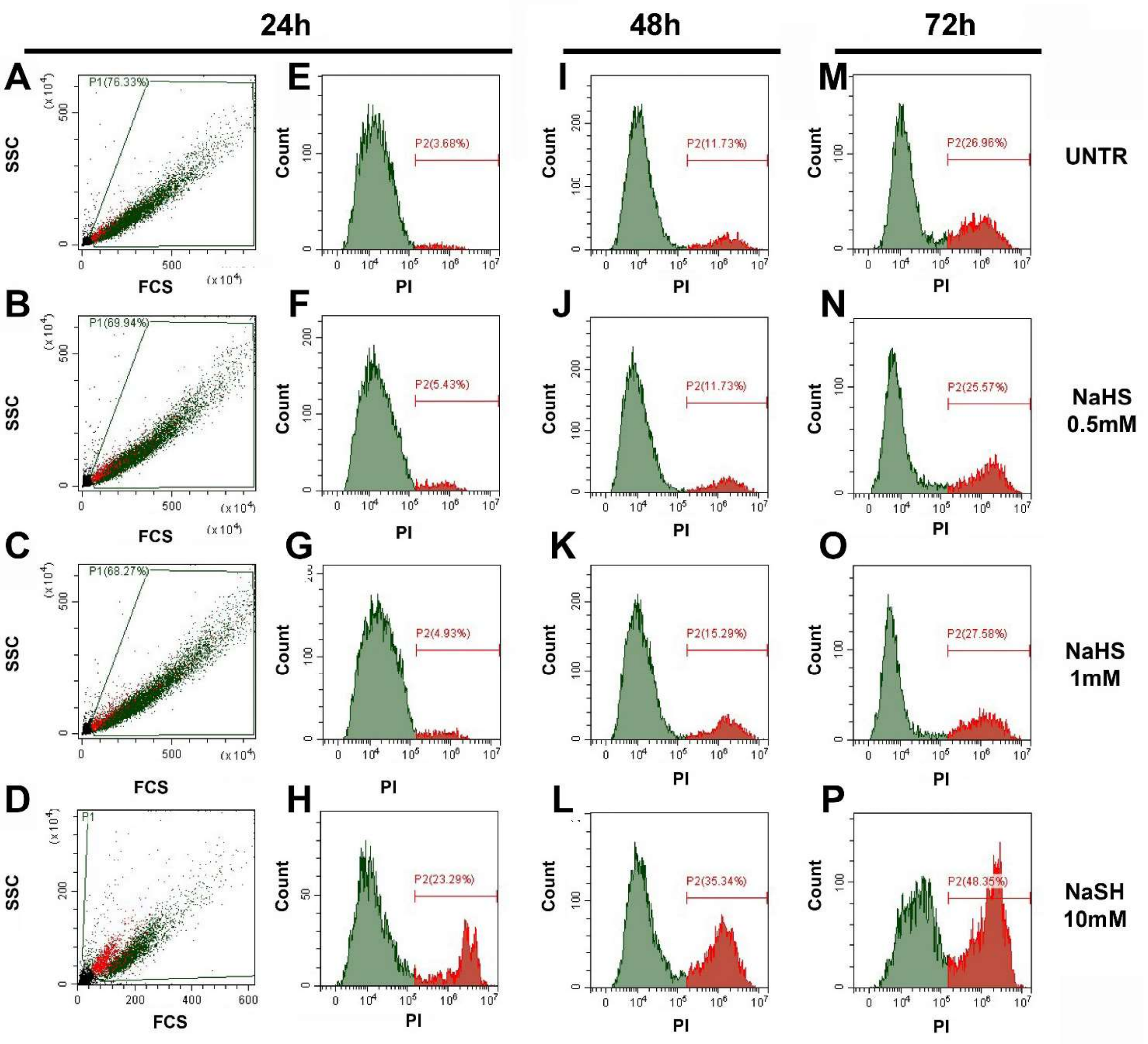

Figure S1: Cell viability on Beas-2B cell line treated with NaHS. Panels A-D are representative dot plots of Forward scatter (FS) and side scatter (SS) parameters in Beas-2B cells cultured for 24h with or without NaHS. Panels E-P are representative plots of Propidium Iodide fluorescence in Beas-2B cells cultured for 24h (panels E-H), 48h (panels I-L) and 72h (panels M-P) without NaHS (panels E, I, M), or different doses of NaHS (Panels F, J, N, G, K, O, H, L, P). The highest dose of NaHS 10mM induces toxic effect, % of PI^+ cells is 23.29%, 32.34% and 48.35% at 24h, 48h, 78h respectively. On the contrary, no toxic effects are reported with NaHS 0.5mM and NaHS 1mM at each time point as compared to untreated cells (% PI^+ cells are: 24h: UNT= 3.69%, NaHS 0.5mM=5.43%, NaHS 1Mm= 4.93%; 48h: UNT=11.73% , NaHS 0.5mM=11.73%, NaHS 1mM=15.29%; 72h: UNT=26.96% , NaHS 0.5mM=25.57%, NaHS 1mM=27.58%).

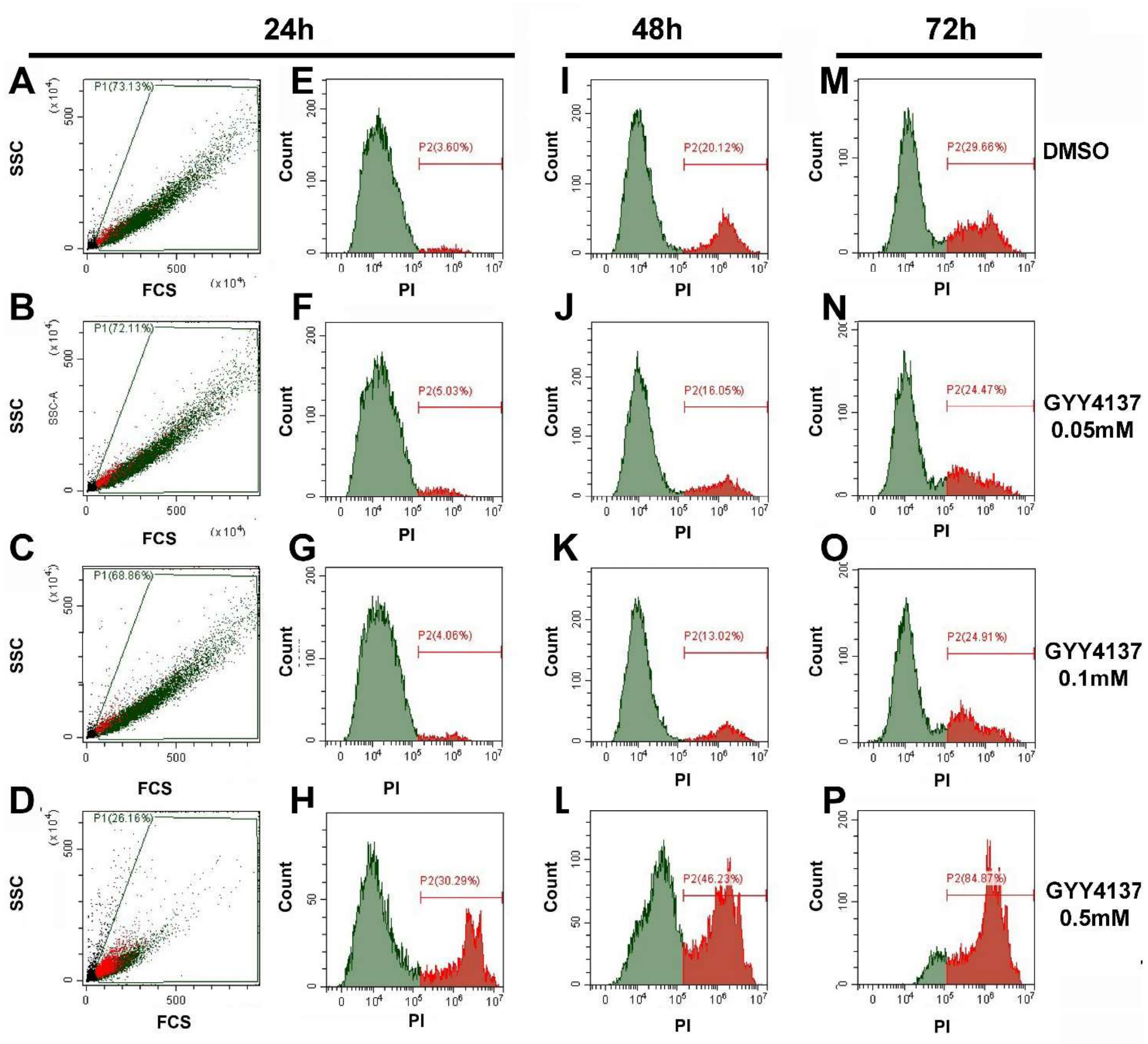

Figure S2: Cell viability on Beas-2B cell line treated with GYY4137. Panels A-D are representative dot plots of Forward scatter (FS) and side scatter (SS) parameters in Beas-2B cells cultured for 24h with DMSO or GYY4137. Panels E-P are representative plots of Propidium Iodide fluorescence in Beas-2B cells cultured for 24h (panels E-H), 48h (panels I-L) and 72h (panels M-P) with DMSO (panels E, I, M), or different doses of GYY4137 (Panels F, J, N, G, K, O, H, L, P). The highest dose of GYY4137 0.5mM induces toxic effect, % of PI^+ cells is 30.29%, 46.23% and 84.87% at 24h, 48h, 78h respectively. On the contrary, no toxic effects are reported with GYY4137 0.05mM and GYY4137 0.1mM at each time point as compared to untreated cells (% of PI^+ cells are: 24h: DMSO= 3.6%, GYY4137 0.05mM=5.03%, GYY4137 0.1Mm= 4.06%; 48h: DMSO=20.12% , GYY4137 0.05mM =16.05%, GYY4137 0.1mM =13.02%; 72h: DMSO=29.66% , GYY4137 0.05mM=24.47%, GYY4137 0.1mM=24.91%).

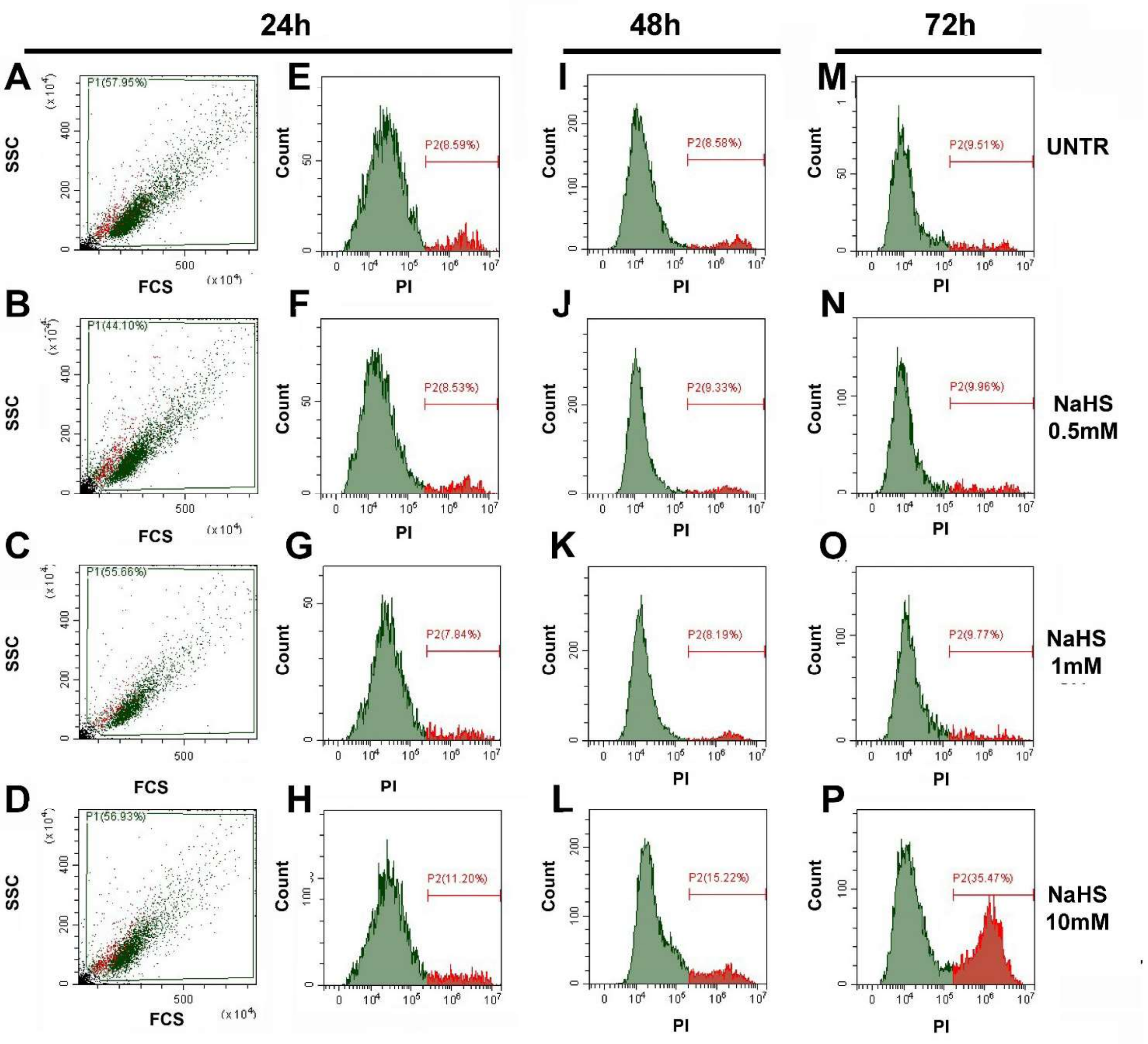

Figure S3: Cell viability on Calu-1 cell line treated with NaHS. Panels A-D are representative dot plots of Forward scatter (FS) and side scatter (SS) parameters in Calu-1 cells cultured for 24h with or without NaHS. Panels E-P are representative plots of Propidium Iodide fluorescence in Calu-1 cells cultured for 24h (panels E-H), 48h (panels I-L) and 72h (panels M-P) without NaHS (panels E, I, M), or different doses of NaHS (Panels F, J, N, G, K, O, H, L, P). The highest dose of NaHS 10mM at 72h of treatment induces toxic effect with 35.47 % of PI^+. On the contrary, no toxic effects are reported with NaHS 0.5mM and NaHS 1mM at each time point as compared to untreated cells (% PI^+ cells are: 24h: UNT= 8.59%, NaHS 0.5mM=8.53%, NaHS 1Mm= 7.84%; 48h: UNT=8.58% , NaHS 0.5mM=9.33%, NaHS 1mM=8.19%; 72h: UNT=9.51% , NaHS 0.5mM=9.96%, NaHS 1mM=9.77%).

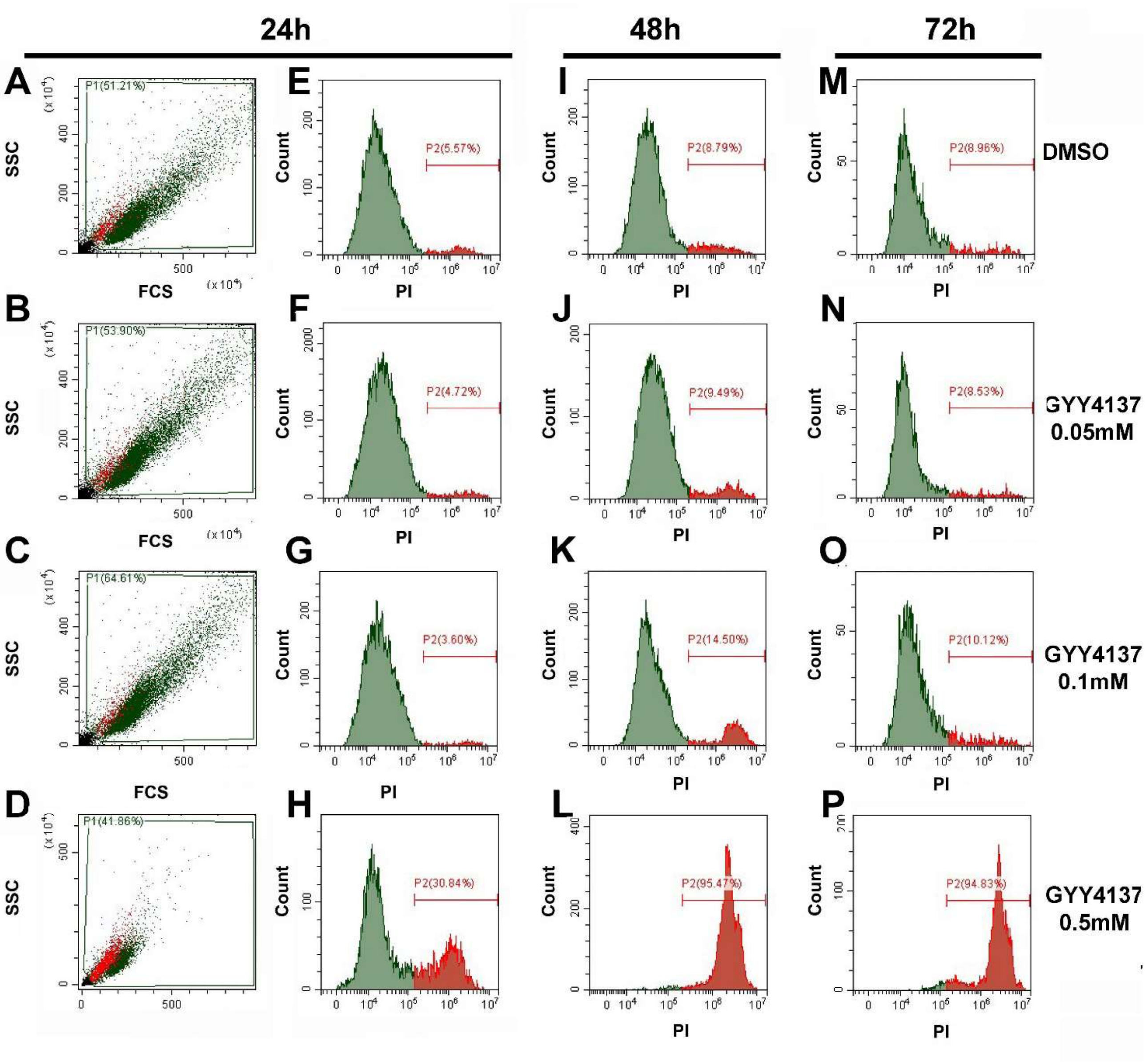

Figure S4: Cell viability on Calu-1 cell line treated with GYY4137. Panels A-D are representative dot plots of Forward scatter (FS) and side scatter (SS) parameters in Calu-1 cells cultured for 24h with DMSO or GYY4137. Panels E-P are representative plots of Propidium Iodide fluorescence in Calu-1 cells cultured for 24h (panels E-H), 48h (panels I-L) and 72h (panels M-P) with DMSO (panels E, I, M), or different doses of GYY4137 (Panels F, J, N, G, K, O, H, L, P). The highest dose of GYY4137 0.5mM induces toxic effect, % of PI^+ cells is 30.84%, 95.47% and 94.83% at 24h, 48h, 78h respectively. On the contrary, no toxic effects are reported with GYY4137 0.05mM and GYY4137 0.1mM at each time point as compared to untreated cells (% of PI^+ cells are: 24h: DMSO= 5.57%, GYY4137 0.05mM=4.72%, GYY4137 0.1Mm= 3.6%; 48h: DMSO=8.79%, GYY4137 0.05mM=9.49%, GYY4137 0.1mM=14.5%; 72h: DMSO=8.96%, GYY4137 0.05mM=8.53%, GYY4137 0.1mM=10.12%).

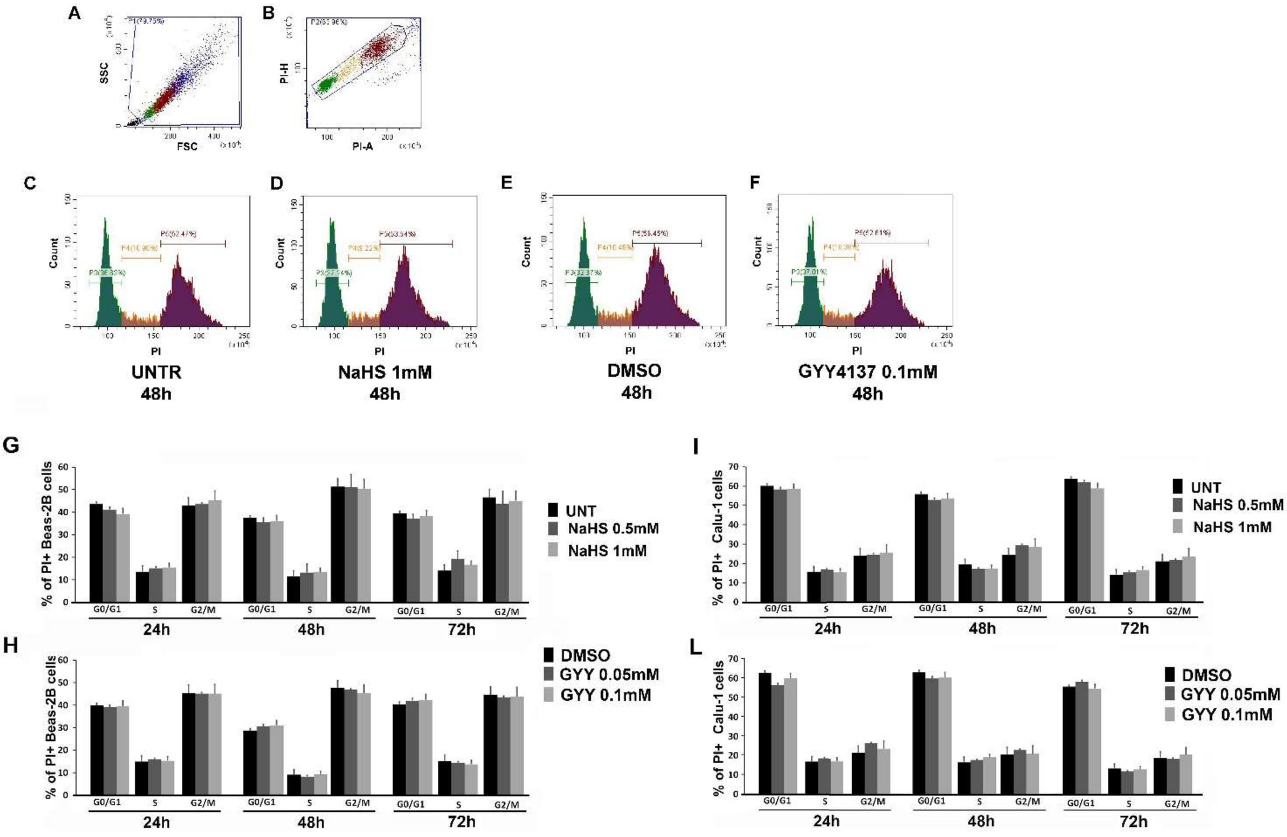

Figure S5: Cell cycle analysis on Beas-2B and Calu-1 cell line. Panels A-F: representative flow cytometric plots of cell cycle analysis of control cells (panels C and E), cells treated with NaHS 1mM (panel D) or GYY4137 0.1mM (panel F) labelled with propidium Iodide (PI) (after cell permeabilization). Panels G-L: percentage of cells in G0/G1, S and G2/M phases reported as mean±SD of at least 3 independent experiments. Beas-2B cells treated with or without NaHS (panel G), Beas-2B cells treated with DMSO or GYY4137 (panel H) and Calu-1 cells treated with or without NaHS (panel I), Calu-1 cells treated with DMSO or GYY4137 (panel L).

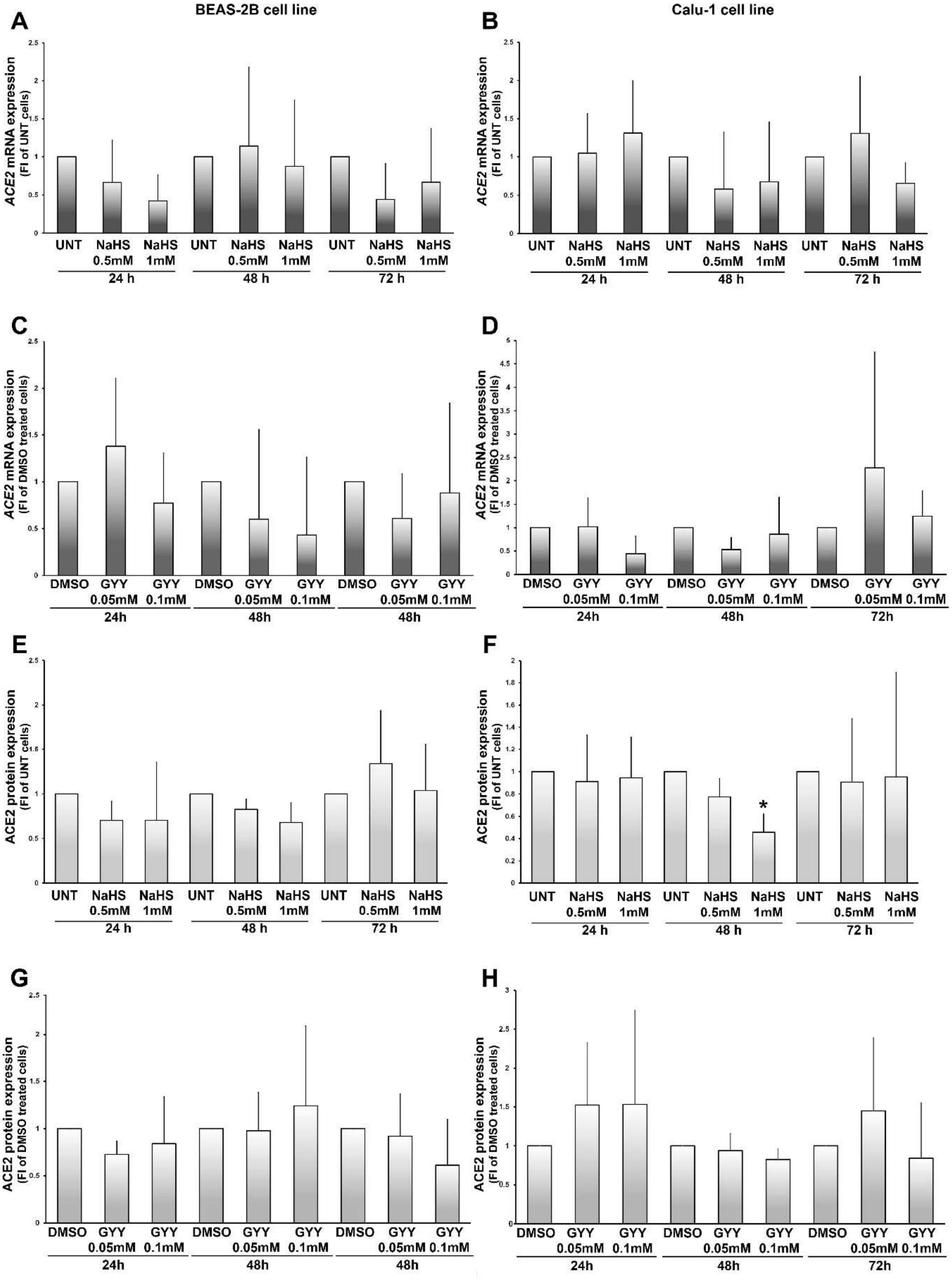

Figure S6: ACE2 expression in BEAS-2B and Calu-1 cells treated with NaHS and GYY4137. Panels A-D: mRNA expression of ACE2 in BEAS-2B (panels A and C) Calu-1 (panels B and D) cultured in presence or absence of NaHS (panels A and B) or GYY4137 (panels C and D) at 24, 48, 72 hours. ACE2 mRNA expression has been normalized to rRNA18S expression for each experimental condition, and data are reported as fold increase of UNT (panels A and B) or DMSO treated (panels C and D) cells for each time point. Panels E-H: protein expression of ACE2 in BEAS-2B (panels E and G)

and Calu-1 (panels F and H) cultured in presence or absence of NaHS (panels E and F) or GYY4137 (panels G and H) at 24, 48, 72 hours. ACE2 protein expression has been normalized to GAPDH expression for each experimental conditions and data are reported as fold increase of UNT (panels E and F) or DMSO treated (panels G and H) cells for each time point. Data are presented as means ± SD of at least 3 independent experiments (*, $p < 0.05$; One-way ANOVA and Dunnett's test).
